# Do older adults with multimorbidity prefer institutional care than those without multimorbidity? The role of functional limitation

**DOI:** 10.1186/s12877-022-02812-2

**Published:** 2022-02-14

**Authors:** Dan Zhao, Jie Li, Tingting Gao, Jingjie Sun, Yi Wang, Qiong Wang, Chengchao Zhou

**Affiliations:** 1 Centre for Health Management and Policy Research, School of Public Health, Cheeloo College of Medicine, Shandong University, Jinan, 250012 China; 2 Shandong Health Commission Medical Management Service Center, Jinan, 250012 China; 3 NHC Key Lab of Health Economics and Policy Research, Shandong University, 250012 Jinan, China

**Keywords:** Long-term care, Institutional care, Willingness, Multimorbidity, Functional dependence

## Abstract

**Background:**

Population ageing and social transformation present tremendous challenges to the informal support system of older adults, which engendered institutional care in China. This study aimed to examine the association between multimorbidity and institutional care willingness, and investigate whether there is an interaction effects between multimorbidity and functional limitations on institutional care willingness among Chinese older adults.

**Methods:**

Data were obtained from the sixth National Health Service Survey of Shandong province, China. The sample included 8583 older adults (age ≥ 60 years; 51.7% women), 44.8% without chronic diseases, 34.8% and 20.4% with one chronic condition and multimorbidity, respectively. Multivariable logistic regression models and marginal effects analysis were used to the interaction effects analysis.

**Results:**

A total of 666 (7.8%) participants had institutional care willingness in Shandong, China. Participants with multimorbidity were more likely to have institutional care willingness than their peers without chronic condition (OR = 1.25, 95% CI = 1.06, 1.55) after adjusted for confounders. Marginal effect analysis showed that under the condition that other variables remain unchanged, the probability of them with multimorbidity choosing institutional care for older adults with functional limitations was 6.9% lower than those without multimorbidity (95% CI = -0.128, -0.010, *P* = 0.023). The interaction effect between chronic health conditions and functional limitation for older adults to choose institutional care was statistically significant, and the average interaction effect was 4.83% (*Z* = -2.70, ${S}_{\overline{x} }$ = 0.0189, *P* < 0.05).

**Conclusions:**

This relationship between multimorbidity and institutional care willingness varied by functional limitations. To better meet the care needs among older adults with multimorbidity and functional limitations, more resources and incentives should be provided to encourage the building-up of eldercare institutions. The governments should also establish long-term care system and to provide better home-based care for older adults, as older adults who prefer home care remain the majority.

**Supplementary Information:**

The online version contains supplementary material available at 10.1186/s12877-022-02812-2.

## Introduction

China has the largest aging population and it grows fastest in the world, stepping into an ultra-aging society. According to The Seventh National Census in 2020, the population aged 60 years and above was 264.02 million, accounting for 18.7% of the total population [1]. It is estimated that aging population will exceed 400 million by 2041 in China [2]. This burgeoning aging population reduced the supply of labor force and increased the burden of family care, posing an unprecedented challenge to the development of long-term care system. Eldercare in China relies heavily on informal care provided by family members due to filial piety [3]. Being filial and respectful to the older adults has been a traditional virtue of the Chinese nation. Filial piety is the core of Confucianism that emphasizes adult children’s respect and care for their parents [4]. However, the shrinking younger generation and the booming aging population are intensifying the pressure on family-based care [5]. This increasing elderly dependency ratio have engendered new ways of social care outside traditional informal care to cope with the consequent surge in demand for eldercare [6].

Institutional eldercare is one of the formal long-term social care service in which social institutions are usually provides their care [7]. Compared with home-based care, institutional care is more conducive to taking advantage of its scale effect and providing professional medical care and nursing services for older adults, especially those with poor health status [8, 9]. Professional eldercare institutions are playing an indispensable role in the long-term care system [10]. Institutional care willingness is defined as the attitude and preference of older adults towards institutional eldercare [11]. It has been demonstrated to be one of the most significant factors that the government should consider when allocating care resources for older adults [12, 13]. Clarifying factors affecting the willingness to receive institutional care is critical to the construction and development of future social care system.

Recently, gerontology research concerning chronic conditions has shifted from single disease type to multimorbidity that was defined as the co-occurrence of at least two chronic diseases [14–16]. It was estimated that 46% of older adults in China are suffering from multimorbidity [17]. Compared with older adults with a single disease, those with multimorbidity are considered to have poorer quality of life, more health service utilization and higher risk of mortality, imposing a heavy economic burden on healthcare system [14, 18]. Most available studies about the correlation between chronic diseases and institutional care willingness have focused on whether older adults suffered from chronic diseases(yes/no), rather than multimorbidity (multi-category). Some Chinese researches on the association between chronic diseases the willingness to receive institutional care in older adults were carried out and those researchers got a positive conclusion [19]. Nihtilae et al. [20] found that chronic medical conditions raised the risk of long-term institutionalization by 50% or more. The latest few studies, however, did not support the association between the multimorbidity and institutional care willingness [21–23]. For example, studies in urban empty nesters and disabled older adults reported that the number of chronic diseases was not related to institutional care willingness [21–23]. Therefore, the relationship between chronic conditions and institutional care willingness is still unclear with controversial results among older adults in China.

Physical functioning decline is common among older adults [24]. Previous evidence have suggested that the quantity of chronic conditions closely associated with functional limitations among older adults [25, 26]. Compared with peers, older adults with functional limitations had the most urgent need for care services [27]. Previous research indicated that social care services focusing on institutional eldercare would be the preferred form of care for disabled older adults in the future [28]. Although disabled older adults have become the main target group for institutional eldercare, research results on the association between functional limitations and institutional care willingness were still controversial. Chen et al. [19] referred that older adults with functional limitations were more likely to choose institutional care in Jiangsu province, China. While another study in Beijing, the capital of China, found that non-disabled elderly also had stronger institutional care willingness [29]. Moreover, it is largely unknown whether the linkage between multimorbidity and institutional care willingness varies across different functional limitations groups.

To further explore these relationships, the aims of this study are as follows: 1) to explore the association between multimorbidity and institutional care willingness among the older adults; 2) to examine whether there is an interaction effect between multimorbidity and functional limitations on institutional care willingness. The study will highlight the degree of willingness for institutional care among people with various levels of disease burden in China, and identify whether greater attention is required to meet the needs of those living with functional limitations. We hypothesize that older adults with multimorbidity would prefer institutional care than their peers without chronic condition, and the relationship between multimorbidity and institutional care willingness varied by functional limitations.

## Methods

### Study design and setting

The data in this study were derived from the sixth Health Service Survey of Shandong province, China in 2018, which is included in the National Health Service Survey (NHSS). NHSS is a nationally representative survey conducted by the National Health Commission every five years since 1993 to fully understand residents’ health and health service needs in China [30]. According to Health Commission of Shandong Province in 2020, population aged 60 or older reached 21.22 million, accounting for 20.90% of the total population. A multistage cluster sampling method was used for selecting the participants. Firstly, 20 counties were randomly selected from 137 counties in Shandong province. Secondly, five townships were randomly selected from each county and two sample villages (communities) were randomly selected from each township. Thirdly, 60 or more households were investigated in each sample village. In total, 100 townships and 200 villages were selected, 12,938 households and 35,264 individuals were included.

### Sample selection

Data were collected from September to October 2018 using face-to-face interviews. Well-trained investigators interviewed all members of the families using structured questionnaires. Given our focus on older adult sample, we restricted our analysis to respondents aged 60 years or older. We excluded 261 participants with dementia and 59 participants without information of institutional care willingness from the total of 8,903 older adults interviewed. Finally, 8,583 older adults were included in this study. Fig. S1 presented a flowchart of the study sample.

### Measurement

#### Institutional care willingness

The dependent variable was the willingness to institutional care in older adults, and assessed by the question “Which way of elder care are you willing for?” If the answer was ‘institutional care’, it was coded as ‘yes’. Conversely, if older adults chose the option of ‘home-based care’ or ‘community-based care’, it was categorized as ‘no’. Willingness here implies an absolute preference for institutional care as opposed to home or community-based care.

#### Chronic diseases and multimorbidity

Data on multimorbidity were mainly collected by patient self-report. Older adults were considered to have chronic diseases based on any of occurrences as follows: (1) There were chronic diseases that have been clearly diagnosed by physician over the past six months, including chronic infectious diseases (such as tuberculosis, etc.) and chronic non-infectious diseases (such as coronary heart disease, hypertension, etc.); (2) Older adults had chronic diseases diagnosed by physician six months ago, and treatment measures (medication or physical therapy) were taken to control the disease within six months before the investigation. Here, the last six months refers to the time when the doctor diagnosed rather than the length of time the participant has suffered from a chronic disease. The National Health Commission prepared a NHSS Disease Classification-Code List for help, including 132 diseases classified into 20 categories. A list of 67 chronic conditions that were eligible to be included in counts was provided in Supplementary Table 1. Multimorbidity was defined as the simultaneous presence of at least two chronic diseases. A simple count of conditions was used to measure multimorbidity. We operationalized chronic health conditions as a variable with three categories based on the number of chronic diseases (no chronic condition, one chronic condition, multimorbidity).

#### Functional limitations

Participants were asked to answer the question about whether they have difficulty in eight self-care activities, including six activities of daily living (ADL) and two instrumental activities of daily living (IADL): a) feeding; b) dressing; c) bathing; d) going to toilet; e) transferring; f) bladder and bowel control; g) doing housework; h) managing money [31, 32]. Response options were ‘without difficulty’, ‘with difficulty but still can complete independently’, ‘with difficulty and need help’ and ‘unable to complete’. Individuals ‘with difficulty and need help’ and ‘unable to complete’ were considered to be dependent. Functional limitations were indicated by dependence in at least one IADL or ADL items. The Chinese version was used to assess the functional limitations of participants, which has been proven to have good reliability and validity [33].

#### Control variables

Previous evidence highlighted other relevant factors for institutional care willingness which were included as covariates in our study [34–38]. We divided these control variables into three categories based on the Andersen model, which was widely used in the field of medical and health services [39, 40]. 1) Predisposing variables, including gender (male, female), age, education (illiteracy, primary school, junior school, middle school or above), marital status (single, married) and employment status (employed, retired, unemployed). 2) Enabling variables, including region (urban, rural), living arrangements (alone, with others), household income in the last year (Q1, Q2, Q3 and Q4), establishment of health records (yes, no), family doctor contract service (yes, no), social activity engagement (yes, no). Economic status was estimated by household income in the last year. It was divided into four types based on quartile. Q1 was the poorest and Q4 was the richest. 3) Need variables, including need caregiving (yes, no) and body mass index (underweight, normal, overweight, obesity). Body mass index (BMI) equals body weight (kg)/height2 (m2). According to the Working Group on Obesity in China [41], appropriate cutoff points of BMI recommended for Chinese adults were as follows: underweight (< 18.5), normal (18.5–24.0), overweight (24.0–28.0) and obesity (> 28.0).

### Statistical analysis

All statistical analyses were performed using Stata 14.0 (Stata Corp, College Station, TX, USA). Student’s t-tests, chi-square tests and Yate’s corrected chi-square test were used to compare the institutional care willingness across different subgroups by functional limitations. Logistic regression models were used to assess the association between multimorbidity and institutional care willingness among older adults. Model 1 included univariate analysis of independent variables and all confounding variables. In model 2, odds ratios (ORs) were shown adjusted for all predisposing, enabling and need variables. In model 3, we further included the interaction term of chronic health conditions and functional limitations. In addition, marginal effect analysis was employed to illustrate the prediction of institutional care willingness by ADL limitations and chronic health conditions. Because an interaction effect in logistic regression is a nonlinear marginal effect whose value depends on the values assumed by all model variables, we next use the computation method of Ai and Norton [42] and Norton et al. [43] to check the sign and significance of the true interaction effects across observations. The reported confidence intervals (CIs) were calculated at the 95% level. *P* values less than 0.05 were considered statistically significant.

## Results

### Characteristics of participants

Table 1 shows that of all respondents, 666 had institutional care willingness. Compared with older adults without institutional care willingness (92.2%), those with institutional care willingness (7.8%) had a lower proportion of females, younger, more education and higher rates of employment. A total of 3,842 (44.8%) were free from chronic diseases, 2,988 (34.8%) and 1,753 (20.4%) had one chronic condition and multimorbidity, respectively. More information of the participants’ characteristics was shown in Table 1.

**Table 1:** Descriptive statistics among older adults in Shandong, China, 2018 (*N* = 8583)

Variables	Institutional care willingness	
	Yes	No
n (%)	666 (7.8)	7917 (92.2)
Gender		
Male	350 (52.6)	3792 (47.9) ^*^
Female	316 (47.4)	4125 (52.1)
Age (Mean ± SD)	66.5 ± 5.6	68.8 ± 6.8 ^***^
Education		
Illiteracy	106 (15.9)	2539 (32.1) ^***^
Primary school	211 (31.7)	2580 (32.6)
Junior school	217 (32.6)	1799 (22.7)
Middle school or above	132 (19.8)	999 (12.6)
Marital status		
Single^a^	93 (14.0)	1287 (16.3)
Married	573 (86.0)	6630 (83.7)
Employment status		
Employed	207 (31.1)	2304 (29.1) ^***^
Retired	214 (32.1)	1819 (23.0)
Unemployed	245 (36.8)	3794 (47.9)
Region		
Urban	283 (42.5)	3901 (49.3) ^***^
Rural	383 (57.5)	4016 (50.7)
Living arrangements		
Alone	74 (11.1)	881 (11.1)
With others	592 (88.9)	7036 (88.9)
Household income^b^		
Q1	163 (24.5)	2030 (25.6)^**^
Q2	176 (26.4)	2340 (29.6)
Q3	154 (23.1)	1956 (24.7)
Q4	173 (26.0)	1591 (20.1)
Health records		
Yes	517 (77.6)	6458 (81.6) ^**^
No	149 (22.4)	1459 (18.4)
Family doctor		
Yes	344 (51.7)	5394 (68.1) ^***^
No	322 (48.3)	2523 (31.9)
Social activity		
Yes	137 (20.6)	1658 (20.9)
No	529 (79.4)	6259 (79.1)
Need caregiving		
Yes	56 (8.4)	805 (10.2)
No	610 (91.6)	7112 (89.8)
BMI		
Underweight	27 (4.1)	466 (5.9) ^*^
Normal	277 (41.6)	3559 (45.0)
Overweight	258 (38.7)	2852 (36.0)
Obesity	104 (15.6)	1040 (13.1)
Chronic health conditions		
No chronic condition	287 (43.1)	3555 (44.9)
One chronic condition	228 (34.2)	2760 (34.9)
Multimorbidity	151 (22.7)	1602 (20.2)
Functional limitations		
Yes	60 (9.0)	849 (10.7)
No	606 (91.0)	7068 (89.3)

^***^ *P* < 0.001; ^**^ *P* < 0.01; ^*^ *P* < 0.05

^a^ Singles include those who are unmarried (81, 0.94%), divorced (51, 6.0%) and widowed (1248, 14.54%)

^b^ Quartile 1 (Q1) was the poorest and Quartile 4 (Q4) was the richest

### Association between multimorbidity and institutional care willingness

Table 2 illustrates that the associations between different chronic health conditions and institutional care willingness among older adults in China. In Model 1, univariate analysis showed that the relationship between multimorbidity and institutional care willingness may be weakened or obscured (OR = 1.17, 95% CI: 0.95, 1.43). It is possible that including some variables with confounding effect may make other variables significant. There may be interactions between independent variables. Model 2 reveals that older adults with multimorbidity (OR = 1.25, 95% CI: 1.06, 1.55) were more likely to have institutional care willingness than their peers without chronic condition after adjusting for potential covariates.

**Table 2:** Association between multimorbidity and institutional care willingness among older adults in Shandong, China, 2018 (*N* = 8583)

Characteristics	Model 1^a^			Model 2^b^			Model 3^c^	
	OR (95% CI)	*P*-value		OR (95% CI)	*P*-value		OR (95% CI)	*P*-value
*Main terms*								
Chronic health conditions (No chronic condition ^Ref^)								
One chronic condition	1.02 (0.85, 1.23)	0.803		1.08 (0.89, 1.30)	0.440		1.09 (0.90, 1.32)	0.396
Multimorbidity	1.17 (0.95, 1.43)	0.104		1.25 (1.06, 1.55)	0.040		1.42 (1.13, 1.78)	0.003
Functional limitations (No ^Ref^)								
Yes	0.82 (0.63, 1.08)	0.168		1.11 (0.80, 1.55)	0.526		2.02 (1.18, 3.43)	0.010
*Interaction term*								
(No chronic condition × Without functional limitations ^Ref^)								
One chronic condition × Functional limitations							0.67 (0.34, 1.32)	0.245
Multimorbidity × Functional limitations							0.29 (0.14, 0.61)	0.001
*Controls*								
Gender (Male ^Ref^)								
Female	0.83 (0.71, 0.97)	0.021		1.09 (0.91, 1.30)	0.344		1.09 (0.91, 1.30)	0.358
Age	0.94 (0.93, 0.96)	< .001		0.95 (0.93, 0.96)	< .001		0.95 (0.93, 0.96)	< .001
Education (Illiteracy ^Ref^)								
Primary school	1.96 (1.54, 2.49)	< .001		1.86 (1.45, 2.40)	< .001		1.89 (1.47, 2.43)	< .001
Junior school	2.89 (2.27, 3.67)	< .001		2.41 (1.83, 3.17)	< .001		2.44 (1.85, 3.22)	< .001
Middle school or above	3.16 (2.43, 4.13)	< .001		2.58 (1.87, 3.56)	< .001		2.61 (1.89, 3.60)	< .001
Marital status^d^ (Single ^Ref^)								
Married	1.20 (0.95, 1.50)	0.122		0.92 (0.67, 1.25)	0.573		0.92 (0.68, 1.25)	0.600
Employment status (Employed ^Ref^)								
Retired	1.31 (1.07, 1.60)	0.008		1.37 (1.04, 1.81)	0.023		1.37 (1.04, 1.80)	0.025
Unemployed	0.72 (0.59, 0.87)	0.001		0.96 (0.78, 1.18)	0.678		0.95 (0.77, 1.18)	0.658
Region (Urban ^Ref^)								
Rural	1.31 (1.12, 1.54)	0.001		1.56 (1.29, 1.89)	< .001		1.57 (1.29, 1.90)	< .001
Living arrangements (With others ^Ref^)								
Alone	1.00 (0.78, 1.28)	0.989		1.11 (0.79, 1.56)	0.544		1.12 (0.80, 1.56)	0.528
Household income^e^ (Q1 ^Ref^)								
Q2	0.94 (0.75, 1.17)	0562		0.75 (0.59, 0.95)	0.015		0.75 (0.59, 0.94)	0.014
Q3	0.98 (0.78, 1.23)	0.866		0.69 (0.54, 0.90)	0.006		0.69 (0.54, 0.90)	0.006
Q4	1.35 (1.08, 1.69)	0.008		0.83 (0.62, 1.11)	0.212		0.83 (0.61, 1.11)	0.205
Health records (Yes ^Ref^)								
No	1.28 (1.05, 1.54)	0.012		0.83 (0.67, 1.04)	0.108		0.83 (0.67, 1.04)	0.109
Family doctor (Yes ^Ref^)								
No	2.00 (1.71, 2.35)	< .001		1.85 (1.54, 2.24)	< .001		1.85 (1.54, 2.23)	< .001
Social activity (Yes ^Ref^)								
No	0.99 (0.83, 1.19)	0.947		0.97 (0.79, 1.18)	0.753		0.97 (0.79, 1.18)	0.730
Need caregiving (Yes ^Ref^)								
No	0.81 (0.61, 1.08)	0.147		0.99 (0.71, 1.38)	0.932		1.03 (0.74, 1.43)	0.882
BMI (Underweight ^Ref^)								
Normal	1.34 (0.89, 2.02)	0.155		1.07 (0.73, 1.36)	0.756		1.08 (0.71, 1.63)	0.729
Overweight	1.56 (1.04, 2.35)	0.032		1.09 (0.57, 1.00)	0.698		1.10 (0.72, 1.68)	0.655
Obesity	1.73 (1.11, 2.67)	0.014		1.20 (1.82, 3.50)	0.431		1.21 (1.77, 1.91)	0.408

^a^ Unadjusted univariate model;

^b^ Adjusted multivariate model;

^c^ Adjusted for model 2 criteria and the interaction between chronic health conditions and functional limitations;

^d^ Singles include those who are unmarried (81, 0.94%), divorced (51, 6.0%) and widowed (1248, 14.54%);

^e ^Quartile 1 (Q1) was the poorest and Quartile 4 (Q4) was the richest

### The interaction effects between multimorbidity and functional limitations on institutional care willingness

**Fig. 1:**
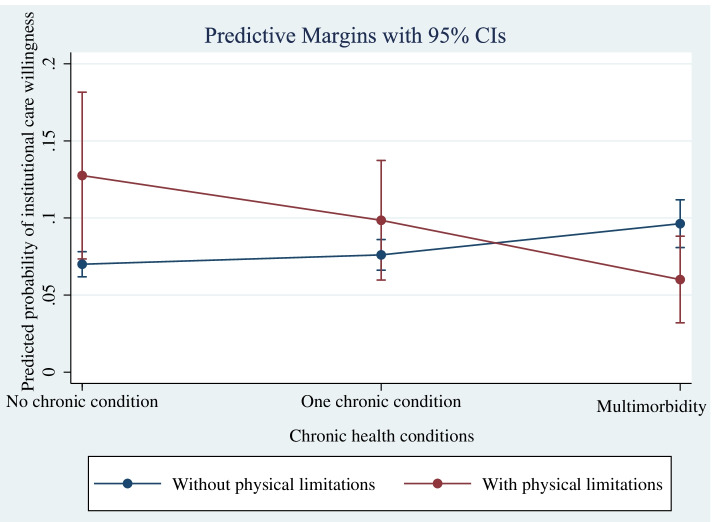
Interaction between multimorbidity and functional limitations in the prediction of institutional care willingness

Further analysis adds the interaction term of chronic health conditions and functional limitations to explore whether the relationship between multimorbidity and institutional care willingness varied by functional limitations in Model 3. Compared with the older adults without chronic condition and functional limitations, those with both dysfunction and multimorbidity had lower institutional care willingness (OR = 0.29, 95% CI: 0.14, 0.61). As shown in Table 3, for older adults with functional limitations, the probability of them with multimorbidity choosing institutional care was 6.9% lower than those without multimorbidity. It showed that when functional limitations was different, the probability of choosing institutional care was different for older adults with different chronic disease conditions. In order to more intuitively show the marginal effect of multimorbidity in different functional limitations, it can be reflected in Fig. 1.

**Table 3:** The marginal effect of the interaction between multimorbidity and functional limitations on the prediction probability of institutional care willingness

Characteristics	*dy/dx*	95%CI	*P*-value
Chronic health conditions			
No chronic condition	(base outcome)		
One chronic condition			
at without functional limitations	0.005	-0.007, 0.018	0.402
at functional limitations	-0.031	-0.095, 0.036	0.350
Multimorbidity			
at without functional limitations	0.026	0.008, 0.043	0.004
at functional limitations	-0.069	-0.128, -0.010	0.023

**Fig. 2:**
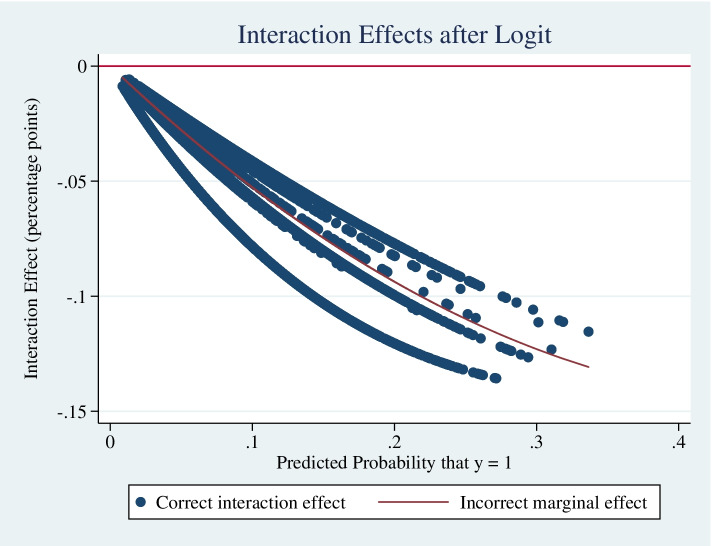
Distributions of interaction effect of chronic health conditions* functional limitation variable on predicted probabilities of institutional care willingness

**Fig. 3:**
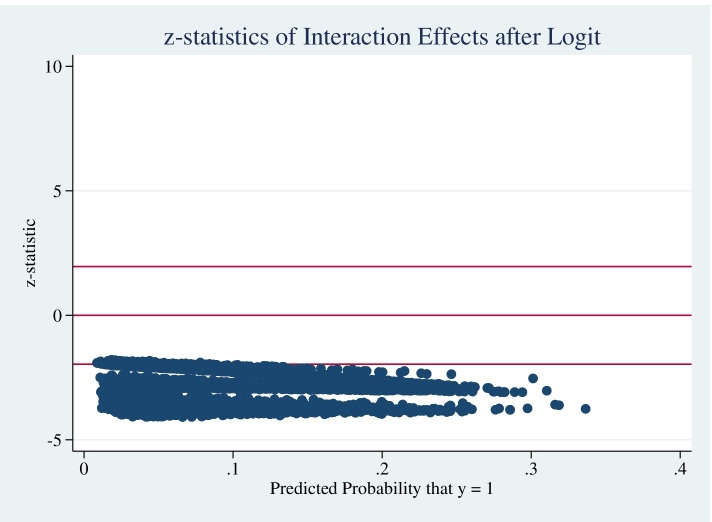
z Statistics plot for interaction effect of chronic health conditions* functional limitation variable on predicted probabilities of institutional care willingness

After using the Stata ‘inteff’ post-estimation command, Table 4 shows that the interaction effect between chronic health conditions and functional limitation for older adults to choose institutional care was statistically significant, and the average interaction effect was 4.83% (*Z* = -2.70, ${S}_{\overline{x} }$=0.0189, *P* < 0.05). Figure 2 plots the interaction term in percentage points against the predicted probability (and the incorrect marginal effect). For all observations the sign of the interaction coefficient was negative, indicating that the coefficients of the significant interaction terms in logistic regressions reflect the true interaction effects reasonably well. Figure 3 reports the z-statistics for the corresponding interaction term, which is statistically significant (*Z* < -1.96, *P* < 0.05).

**Table 4:** Effect of chronic health conditions*functional limitation interaction variable

Variable	N	Mean	Std. Dev	Min	Max
Interaction effect	8,583	-0.0483	0.0251	-0.1357	-0.0058
Interaction standard error	8,583	0.0189	0.0105	0.0018	0.0472
Interaction Z score	8,583	-2.7034	0.6117	-4.0804	-1.7838

## Discussion

To our knowledge, this is the first study to explore the role of functional limitations in the association between multimorbidity and institutional care willingness among Chinese older adults. The vast majority of older adults prefer community or home care however those with multimorbidity were slightly more likely to show institutional care willingness. While in the groups with functional limitations, older adults with multimorbidity had less institutional care willingness.

Our study found that 7.8% older adults were willing to choose institutional care. It was lower than the proportion among the rural elderly in Hunan Province (10.8%) [10] and Jiangsu Province, China (14.2%) [19]. Besides, it was much lower than that in Heilongjiang Province (45.4%) [34], and Chengdu (44.8%), the capital of Sichuan Province, China [44]. The possible reason is the domestic cultural differences. Shandong is a province deeply influenced by the Confucian filial piety culture [37]. Confucianism has a profound influence on Chinese culture, which emphasizes traditional sense of responsibility and filial piety [45]. Adult children have the responsibility to support and take care of their parents to avoid moral condemnation. Older adults also have the idea of “rearing children for old age” and prefer to spend their old age with their children at home. We also found that older adults with higher education are more likely to choose institutional care, which is consistent with previous research [27, 38]. Older adults with higher education level may be more able to accept new ideas and adapt to new environments, and the less they may be affected by traditional pension concepts.

Results from our study indicated that multimorbidity was positively associated with institutional care willingness. Older adults with multimorbidity were slightly more likely to show institutional care willingness than those without multimorbidity. This finding was consistent with previous studies on the relationship between chronic diseases and institutional care willingness[19]. The more types of chronic diseases older adults have, the higher the requirements for care [27]. As Zhang et al. [27] mentioned, traditional home-based care was often difficult to meet the care needs of the older adults with multimorbidity because their demand for eldercare services was high-level and multi-faceted. One possible reason was that eldercare institutions can provide more medical care and rehabilitation care with high technical and professional requirements, which was beneficial for the treatment of multimorbidity [46]. Another reason may be that the miniaturization of the Chinese family structure and the busy work of children weakened the intergenerational family support function [47]. Some older adults with multimorbidity may not want to pose the burden on their children. They prefer institutional care, because the staff of institution could provide prompt treatment when their disease worsens. In addition, older adults with multimorbidity can participate in a variety of leisure activities in institutions, which can help improve their mental health and recovery from chronic conditions [27].

We further revealed that the relationship between multimorbidity and institutional care willingness varied by functional limitations among older adults. For older adults without functional limitations, multimorbidity increases the possibility of institutional care willingness. However, those with the simultaneous presence of both multimorbidity and functional limitations had less institutional care willingness. Actually, previous studies have found that when older adults have ADL limitations, those with poor self-reported health status preferred home-based care instead of institutional care [48]. Several possible explanations for this finding are as follows. First, older adults with both multimorbidity and functional limitations would use much health care services, which may make them impoverished by illness and unable to afford the cost of an eldercare institution [17]. The social health insurance covered more than 95% of the Chinese population in 2013, and the average reimbursement rate was approximately 50%–70%. Nevertheless, financial hardships are still the main reason for not using health care services when necessary [49]. Second, disabled older adults may trust their families more than others due to the emotional fragility and psychological stress, and prefer adult children or relatives to take care of themselves [50–52]. Third, due to the unclear positioning and functions of public eldercare institutions, some of them are less likely to accept older adults with functional limitations based on cost considerations, which may make older adults lose confidence and expectation in institutional care [53]. Thus, functional limitations would decrease the possibility of institutional care willingness among older adults with multimorbidity.

These findings provided some inspiration for the rational layout of institutional care and the improvement of the eldercare system. Firstly, policymaker should ensure institutional care services meet the needs of people with multimorbidity who have functional limitations. Secondly, eldercare institutions should reassure older people and their families that they will receive a high quality institutional care, and work with people with functional limitations and their caregivers to design better services. Last but not least, due to the influence of Confucian filial piety culture, no matter how the elderly care institutions improve their services, some elderly people will still choose home-based care. Therefore, governments should establish long-term care system and to provide better home-based care for older adults.

Several limitations of this study also need to be acknowledged. First, this study was a cross-sectional study and it could only explain the association between multimorbidity and institutional care willingness instead of casual relationships. In future research, longitudinal designs can be used. Second, for the data constraints, the measurement of functional limitations in this study was suboptimal. Although six activities of daily living (ADL) should be the best choice for assessing functional limitations, we added 2 IADL items in order to capture a wider range of functional limitations. Third, institutional care willingness was a dichotomous variable because more specific information was unavailable in the questionnaire. It is more appropriate to use continuous scales instead of dichotomous variables to measure institutional care willingness. Willingness here implies an absolute preference for institutional care as opposed to home or community-based care. Finally, this study was applicable to older adults in Shandong province, China, and other populations need to be verified in future studies.

## Conclusions

In summary, our study suggested the association between multimorbidity and institutional care willingness, and this relationship varied according to functional limitations. To better meet the care needs among older adults with multimorbidity and functional limitations, more resources and incentives should be provided to establish eldercare institutions. The governments should also establish long-term care system and to provide better home-based care for older adults, as older adults who prefer home care remain the majority.

## Supplementary Information

**Additional file 1.** 

## Data Availability

The datasets used in the current study are not publicly available due to the confidential policy but are available from the corresponding author on reasonable request.
