# Supplementary material for: Do older adults with multimorbidity prefer institutional care than those without multimorbidity? The role of functional limitation
Source: BMC Geriatr. 2022 Feb 14;22:126. doi: 10.1186/s12877-022-02812-2 (PMC8845225; doi:10.1186/s12877-022-02812-2)
Supplement: Supplementary file 1 — Additional file 1. [file 12877_2022_2812_MOESM1_ESM.docx]

**Supplementary figures and tables**

**
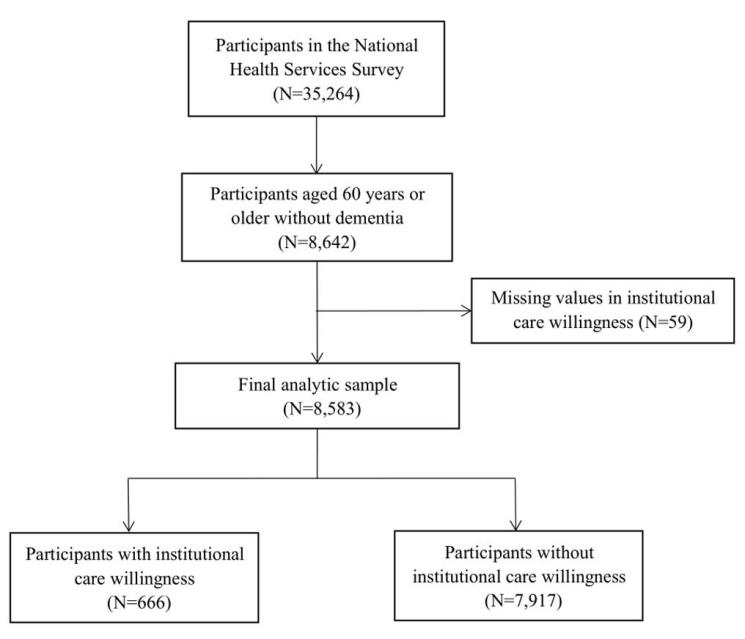
**

Figure S1**.** Flowchart of the study sample.

| **Supplementary Table 1 National Health Service Survey Disease Classification- Code List** | | | |
| --- | --- | --- | --- |
| **Code** | **Disease Name** | **Code** | **Disease Name** |
| *A* | *communicable diseases control* | **067** | **Cerebrovascular disease** |
| 001 | Typhoid and paratyphoid | **068** | **Varicose vein of lower limb** |
| 002 | Bacterial food poisoning | **069** | **Other circulatory diseases** |
| 003 | Dysentery | *L* | *Respiratory diseases count* |
| 004 | Hepatitis A; epidemic jaundice | 070 | Acute nasopharyngitis（The common cold） |
| 005 | Other intestinal infectious diseases | 071 | Acute infection of the upper respiratory tract such as pharynx, larynx, tonsil and trachea |
| 006 | Tuberculosis | 072 | Influenza |
| 007 | Tetanus; lockjaw | 073 | Pneumonia |
| 008 | Blood poisoning; septicemia | **074** | **Chronic pharyngitis, laryngitis** |
| 009 | Measles; hives; morbilli; rubeola | **075** | **Emphysema** |
| 010 | Epidemic encephalitis B | **076** | **Other chronic obstructive pulmonary disease (COPD, including chronic bronchitis)** |
| 011 | Songo fever; epidemic hemorrhagic fever; | **077** | **Asthma** |
| 012 | Hepatitis B | 078 | Other respiratory diseases (including acute lower respiratory infections) |
| 013 | Typhus billosus nostras; leptospirosis | *M* | *Digestive system disease count* |
| 014 | SARS | 079 | Dental disease |
| 015 | Other non-communicable diseases | 080 | Other oral or salivary gland and jaw diseases |
| *B* | *Parasitrometer* | **081** | **Acute and chronic gastroenteritis** |
| 016 | Malaria; ague; impaludism | **082** | **Peptic ulcer** |
| 017 | Bilharziasis;bilharziasis;schistosomiasis;snail fever | **083** | **The appendix disease** |
| 018 | Other parasitic diseases | **084** | **Abdominal hernia** |
| *C* | *Malignancy meter* | 085 | Intestinal obstruction |
| **019** | **Nasopharyngeal malignancy** | **086** | **Chronic liver disease and cirrhosis** |
| **020** | **Esophageal malignant tumor** | **087** | **Gallstones and cholecystitis** |
| **021** | **Gastric malignant tumor** | **088** | **Other digestive diseases** |
| 022 | Malignant tumor of colon | *N* | *Diseases of the urogenital system count* |
| **023** | **Malignant tumors of rectum and anus** | **089** | **Nephritis and nephropathy** |
| 024 | Hepatic malignant tumor | **090** | **Pyelitis** |
| 025 | Pancreatic malignant tumor | **091** | **Urolithiasis** |
| **026** | **Malignant tumors of the trachea, bronchus, and lung** | **092** | **Other urinary diseases** |
| **027** | **Breast cancer** | **093** | **Hyperplasia or inflammation of the prostate** |
| **028** | **Malignant tumor of the cervix** | **094** | **Other diseases of the male reproductive organs** |
| 029 | leukemia； | 095 | Breast disease |
| **030** | **Other malignant tumors** | 096 | Salpingitis and ovaritis |
| *D* | *Benign, in situ and dynamic undetermined tumor meter* | 097 | Uterine vaginal prolapse |
| **031** | **Benign tumor of uterus** | 098 | Other diseases of the female reproductive organs |
| **032** | **Benign brain tumor** | *O* | *Diseases of pregnancy, childbirth and puerperium count* |
| **033** | **Other benign tumors** | 099 | Spontaneous abortion |
| 034 | tumor in situ | 100 | * Induced abortion |
| 035 | Tumor subtotals whose dynamics are uncertain or unknown | 101 | Bleeding during pregnancy and childbirth |
| *E* | *Endocrine, nutritional and metabolic diseases and immune diseases* | 102 | Hypertensive syndrome of pregnancy |
| **036** | **Hyperthyroidism** | 103 | * Normal delivery |
| **037** | **Diabetes; diabetes mellitus** | 104 | Obstructive labor |
| 038 | Lack or poor nutrition | 105 | Puerperium symptoms |
| 039 | Ahmadinejad: Rickets | 106 | Other diseases of pregnancy and childbirth and puerperium complications |
| **040** | **Obesity and other nutritional excesses** | *P* | *Diseases of the skin and subcutaneous tissue count* |
| **041** | **Other internal, camp, generational and immune diseases** | **107** | **Carbuncle and furuncle** |
| *F* | *Blood and hematopoietic organ disease meter* | **108** | **Skin inflammation** |
| **042** | **Anemia** | **109** | **Other diseases of the skin and subcutaneous tissues** |
| **043** | **Other diseases of the blood and hematopoietic organs** | *Q* | *musculoskeletal system and connective tissue disorders count* |
| *G* | *Psychiatric meter* | **110** | **Rheumatoid arthritis** |
| **044** | **Old age, early organic psychosis of old age** | **111** | **Intervertebral disc diseases** |
| **045** | **Schizophrenia** | **112** | **Osteomyelitis** |
| **046** | **Depressive disorder;depression** | **113** | **Other motor diseases** |
| **047** | **Other mental disorders** | *R* | *Congenital abnormalities count* |
| *H* | *Neuropathometer* | **114** | **Congenital heart disease** |
| 048 | Meningitis; cephalomeningitis | **115** | **Other congenital abnormalities** |
| **049** | **Epilepsy** | *S* | *conditions originating in the perinatal period count* |
| 050 | Acute infectious polyneuritis | 116 | Premature and immature infants |
| **051** | **Parkinson's disease** | 117 | Birth trauma |
| **052** | **Other neurological disorders** | 118 | Fetal and neonatal asphyxia |
| *I* | *Eye and accessory disease meter* | 119 | Neonatal tetanus |
| **053** | **Glaucoma** | 120 | Other neonatal diseases |
| **054** | **Cataract** | *T* | *Injury and poisoning count* |
| **055** | **Corneal disease meter** | **121** | **Fracture** |
| **056** | **Other diseases of the eye and appendages** | 122 | Dislocation, sprain and strain |
| *J* | *Ear and mastoid disease meter* | **123** | **Intracranial and internal lesions (including nerves)** |
| **057** | **Otitis media and mastoiditis** | 124 | Open trauma and vascular injury |
| **058** | **Other ear and mastoid diseases** | 125 | Burn |
| *K* | *Circulatory system disease meter* | 126 | Poisoning and toxic effects |
| 059 | Acute rheumatic fever | 127 | Other injuries and poisoning |
| **060** | **Chronic rheumatic heart disease** | *V* | *Others* |
| **061** | **Angina;stenocardia;angor pectoris** | 128 | Pregnancy monitoring |
| 062 | Acute myocardial infarction | 129 | Sterilization |
| **063** | **Other ischemic heart disease** | 130 | Hospitalized for special treatment |
| **064** | **Cor pulmonale** | 131 | Personal and crowd inspection |
| **065** | **Other types of heart disease** | 132 | Other reasons |
| **066** | **Hypertension** | *999* | *Signs, symptoms and ambiguous situation* |

*Note:* *Not counted as a disease. The diseases in bold are chronic diseases included in this study.

| **Supplementary Table 2 Collinearity Diagnostics** | | | | |
| --- | --- | --- | --- | --- |
| Variable | VIF | SQRT VIF | Tolerance | R-Squared |
| Chronic health conditions | 1.09 | 1.04 | 0.9204 | 0.0796 |
| Functional limitations | 1.45 | 1.20 | 0.6888 | 0.3112 |
| Gender | 1.23 | 1.11 | 0.8134 | 0.1866 |
| Age | 1.28 | 1.13 | 0.7798 | 0.2202 |
| Education | 1.51 | 1.23 | 0.6605 | 0.3395 |
| Marital status | 1.80 | 1.34 | 0.5562 | 0.4438 |
| Employment status | 1.30 | 1.14 | 0.7671 | 0.2329 |
| Region | 1.15 | 1.07 | 0.8712 | 0.1288 |
| Living arrangements | 1.70 | 1.30 | 0.5893 | 0.4107 |
| Household income | 1.44 | 1.20 | 0.6955 | 0.3045 |
| Health records | 1.41 | 1.19 | 0.7097 | 0.2903 |
| Family doctor | 1.43 | 1.20 | 0.6979 | 0.3021 |
| Social activity | 1.05 | 1.02 | 0.955 | 0.045 |
| Need caregiving | 1.38 | 1.17 | 0.7268 | 0.2732 |
| BMI | 1.08 | 1.04 | 0.9251 | 0.0749 |
| *Note：*Mean VIF=1.35 |  |  |  |  |
